# Enhancing Production of Bio-Isoprene Using Hybrid MVA Pathway and Isoprene Synthase in *E. coli*

**DOI:** 10.1371/journal.pone.0033509

**Published:** 2012-04-27

**Authors:** Jianming Yang, Mo Xian, Sizheng Su, Guang Zhao, Qingjuan Nie, Xinglin Jiang, Yanning Zheng, Wei Liu

**Affiliations:** 1 Biomaterials Center, Qingdao Institute of Bioenergy and Bioprocess Technology, Chinese Academy of Sciences, Qingdao, China; 2 Department of Biochemistry, Beijing Risun Chemical Technologies Institute, China Risun Coal Chemicals Group Limited, Beijing, China; 3 College English Office, Foreign Languages School, Qingdao Agricultural University, Qingdao, China; University of Melbourne, Australia

## Abstract

The depleting petroleum reserve, increasingly severe energy crisis, and global climate change are reigniting enthusiasm for seeking sustainable technologies to replace petroleum as a source of fuel and chemicals. In this paper, the efficiency of the MVA pathway on isoprene production has been improved as follows: firstly, in order to increase MVA production, the source of the “upper pathway” which contains HMG-CoA synthase, acetyl-CoA acetyltransferase and HMG-CoA reductase to covert acetyl-CoA into MVA has been changed from *Saccharomyces cerevisiae* to *Enterococcus faecalis*; secondly, to further enhance the production of MVA and isoprene, a alanine 110 of the *mvaS* gene has been mutated to a glycine. The final genetic strain YJM25 containing the optimized MVA pathway and isoprene synthase from *Populus alba* can accumulate isoprene up to 6.3 g/L after 40 h of fed-batch cultivation.

## Introduction

Isoprene (2-methyl-1,3-butadiene) was firstly discovered as a cell metabolite in the mid-1950s by Sanadze.[1], [2]. It functions as a thermoprotectant of plant membranes or as an antioxidant [3], [4], or may have a signaling function, altering the flowering time in some plants [5]. As an important platform chemical, isoprene has been used in industrial production of synthetic rubber for tires and coatings [6] or aviation fuel [7].

Isoprene is the progenitor of the isoprenoid family of compounds[8]. Many commercially relevant isoprenoids exist in nature in small quantity and the yield produced from their natural organisms remains rather low. The depletion of fossil sources and the structural complexity of isoprenoids make it difficult or costly to produce isoprenoids by means of chemical synthesis. Isoprene is no exception since it is produced entirely from petrochemical sources through chemical synthesis method [9], [10], [11].

**Figure 1:**
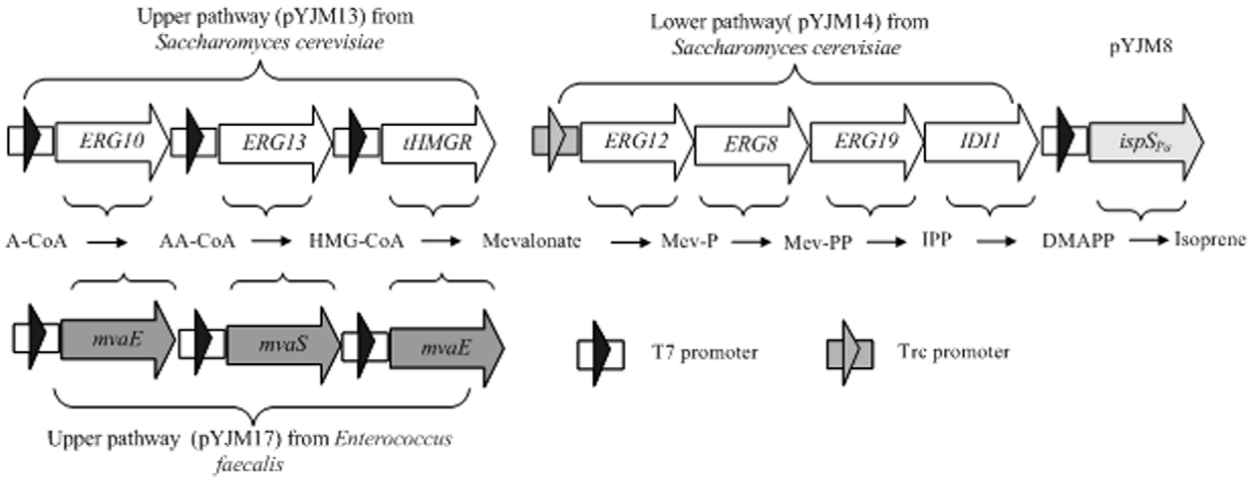
Production of isoprene via the MVA pathways used in this study. Gene symbols and the enzymes they encode (all genes marked with white arrows were isolated from *S. cerevisiae*, the gene marked with light gray arrows derived from *P. alba* and all genes marked with gray arrows were from *Enterococcus faecalis*). MVA pathway: ERG10, acetoacetyl-CoA thiolase; ERG13, HMG-CoA synthase; tHMGR, truncated HMG-CoA reductase; MvaE, acetyl-CoA acetyltransferase/HMG-CoA reductase; MvaS, HMG-CoA synthase; ERG12, mevalonate kinase; ERG8, phosphomevalonate kinase; ERG19, mevalonate pyrophosphate decarboxylase; IDI1, IPP isomerase; ispSPa, *P. alba* isoprene synthase was optimized to the preferred codon usage of *E. coli*. Pathway intermediates. MVA pathway: A-CoA, acetyl-CoA; AA-CoA, acetoacetyl-CoA; HMG-CoA, hydroxymethylglutaryl-CoA; Mev-P, mevalonate 5-phosphate; Mev-PP, mevalonate pyrophosphate. IPP, isopentenyl pyrophosphate; DMAPP, dimethylallyl pyrophosphate.

Compared with conventional means, microbial synthesis of isoprene by fermentation should become a promising and attractive route mainly for environmental production, renewable resources, sustainable development[12]. Additionally, isoprene could be collected from the gas phase of the fermentor, eliminating the need for distillation. All isoprenoids are biosynthesized from the same basic units, isopentenyl diphosphate (pyrophosphate IPP), and its isomer dimethylallyl diphosphate (DMAPP), which are synthesized from two different pathways including methylerythritol 4-phosphate (MEP) pathway and mevalonate (MVA) pathway (Fig.1) [13]. MVA pathway mainly exists in eukaryotes, archaebacteria, and cytosols of higher plants, while the MEP pathway is used by many eubacteria, green algae, and chloroplasts of higher plant [14], [15]. MVA pathway has been studied extensively for producing isoprenoids. The introduction of heterologous MVA pathway genes into E. coli has been reported to improve the productivity of carotenoids or sesquiterpenes that are synthesized from DMAPP[16], [17], [18], [19], [20], [21], [22].

Although MVA pathway has been studied comprehensively, the methylerythritol phosphate (MEP) pathway was merely discovered in the early 1990s by labeling experiments in bacteria and plants[23], [24], and till 2001 the genes of whole MEP pathway has been completely characterized [25]. This biosynthetic pathway, made up of seven enzymatic steps, begins with the condensation of pyruvate and glyceraldehyde-3-phosphate to form 1-deoxy-D-xylulose 5-phosphate (DXP) and ends with the formation of the isoprenoid precursors isopentenyl diphosphate (IPP) and dimethylallyl diphosphate (DMAPP) [26], [27]. In spite of great efforts taken in isoprenoids production using MEP pathway[28], [29], [30], this approach still remains ineffective due to regulation mechanisms present in the native host[18].

In this paper, based on our previous experiments, the efficiency of the MVA pathway on isoprene production has largely been improved as follows: firstly, in order to increase MVA production, the source of the “upper pathway” which contains HMG-CoA synthase, acetyl-CoA acetyltransferase and HMG-CoA reductase to covert acetyl-CoA into MVA has been changed from *Saccharomyces cerevisiae* to *Enterococcus faecalis*; secondly, to further enhance the production of MVA and isoprene, a alanine 110 of the *mvaS* gene has been mutated to a glycine. The final genetic strain YJM25 containing the optimized MVA pathway and isoprene synthase from *P. alba* can accumulate isoprene up to 6.3 g/L after 40 h of fed-batch cultivation, which is approximately a 12-fold increase in isoprene production compared with the previous data [25].

## Materials and Methods

### Bacterial Strains, Plasmids, and Growth Conditions

All Strains and plasmids used in this study are listed in Table 1. *E. coli* strains were grown in LB medium. For MVA or isoprene production, recombinant strains were cultured in shake-flask or fed-batch fermentation with the medium containing glucose 20 g/l, K_2_HPO_4_ 9.8 g/l, beef extract 5 g/l, ferric ammonium citrate 0.3 g/l, citric acid monohydrate 2.1 g/l, MgSO_4_ 0.06 g/l and 1 ml trace element solution which includes (NH_4_)_6_ Mo_7_O_24_·4H_2_O 0.37 g/l, ZnSO_4_·7 H_2_O 0.29 g/l, H_3_BO_4_ 2.47 g/l, CuSO_4_·5H_2_O 0.25 g/l, and MnCl_2_·4H_2_O 1.58 g/l. If necessary, appropriate antibiotics were added to the culture medium at the following concentration: ampicillin (Amp, 100 µg/ml), kanamycin (Kan, 50 µg/ml), and chloramphenicol (Cm, 34 µg/ml).

**Table 1:** Strains, plasmids and Oligonucleotide primers used in this study.

Name	Relevant characteristics	References	
**Strains**			
*E.coli* BL21(DE3)	F^−^ *ompT hsd*S_B_ (r_B_^−^m_B_^−^) *gal dcm rne*131 λ(DE3)	Invitrogen	
*E.coli* JM109(DE3)	*end*A1 *rec*A1 *gyr*A96 *thi hsd*R17 (rk^−^ mk^+^) *rel*A1*sup*E44 Δ(*lac-pro*AB) [F′ *tra*D36*pro*AB^+^ *laq*I^q^ZΔM15] λ(DE3)	TaKaRa	
*E.coli* BL21star^™^ (DE3)	F^−^ *ompT hsd*S_B_ (r_B_^−^m_B_^−^) *gal dcm rne*131 lon λ(DE3)	Invitrogen	
*Saccharomyces cerevisiae*	Type strain	ATCC	
YJM8	*E.coli* BL21(DE3)/pYJM8	[1]	
YJM11	*E.coli* BL21(DE3)/pYJM13	[1]	
YJM12	*E.coli* BL21(DE3)/pYJM8,pYJM14	[1]	
YJM13	*E.coli* BL21(DE3)/pYJM8, pYJM13,pYJM14	[1]	
YJM16	*E.coli* BL21(DE3)/pYJM16	This study	
YJM17	*E.coli* BL21(DE3)/pYJM17	This study	
YJM20	*E.coli* BL21(DE3)/pYJM20,pYJM14	This study	
YJM21	*E.coli* BL21(DE3)/pYJM21, pYJM14	This study	
YJM22	*E.coli* JM109(DE3)/pYJM20,pYJM14	This study	
YJM23	*E.coli* JM109(DE3)/pYJM21,pYJM14	This study	
YJM24	*E.coli* BL21star^™^(DE3)/pYJM20,pYJM14	This study	
YJM25	*E.coli* BL21star^™^ (DE3)/pYJM21,pYJM14	This study	
**Plasmids**			
pACYCDuet-1	P15A (pACYC184), Cm^r^	Novagen	
pCOLADuet™-1	ColA origin, Kan^r^	Novagen	
pTrcHis2B	pBR322 origin, Amp^r^	Invitrogen	
pYJM8	pACYCDuet-1 carrying *ispS_Pa_* from *Populus alba*	[1]	
pYJM13	pCOLADuet™-1 carrying *ERG10, ERG13* and *tHMGR*, from *Saccharomyces cerevisiae*	[1]	
pYJM14	pTrcHis2B carrying *ERG12*, *ERG8*, *ERG19* and *IDI1* from *Saccharomyces cerevisiae*	[1]	
pYJM15	pACYCDuet-1 carrying *mvaE* from *Enterococcus faecalis*	This study	
pYJM16	pACYCDuet-1 carrying *mvaE and mvaS*from *Enterococcus faecalis*	This study	
pYJM17	pACYCDuet-1 carrying *mvaE and mvaS_MT_* from *Enterococcus faecalis*	This study	
pYJM18	pACYCDuet-1 carrying *mvaE* from*Enterococcus faecalis, ispS_Pa_* from *Populus alba*	This study	
pYJM20	pACYCDuet-1 carrying *mvaE and mvaS* from *Enterococcus faecalis, ispS_Pa_* from *Populus alba*	This study	
pYJM21	pACYCDuet-1 carrying *mvaE and mvaS_MT_*from *Enterococcus faecalis, ispS_Pa_* from *Populus alba*	This study	

### Plasmid Construction

Standard DNA manipulations were carried out as previously described by Sambrook *et al*. [31]. Polymerase chain reaction (PCR) was performed using *Pfu* DNA polymerase (TaKaRa, Dalian, China) according to the manufacturer’s instruction.

### Construction of Plasmid for Upper pathway of MVA

**Figure 2:**
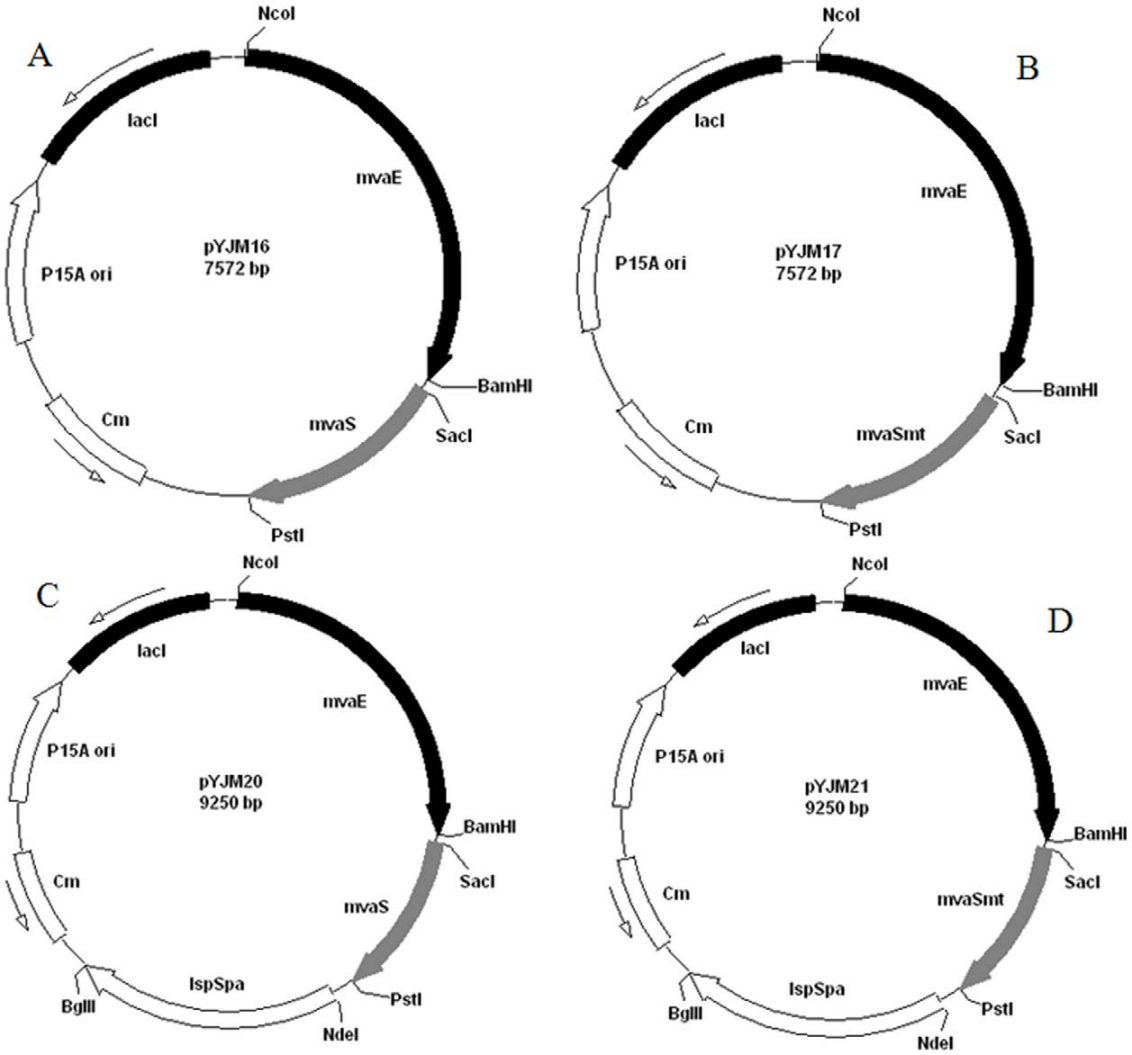
Plasmids used in this study.

The *mvaS* (HMG-CoA synthase, GenBank No. AAG02439) and *mvaE* (acetyl-CoA acetyltransferase/HMG-CoA reductase, GenBank No. AAG02438) genes from *E. faecalis* were chemically synthesized by Genray Company with plasmid pGH as vector (named pGH/mvaS, pGH/mvaE). The *mvaE* was obtained by PCR using the primers mvaE-F (5′-CATGCCATGGAGGAGGTAAAAAAACATGAAAACAGTAGTTATTATTGATGC-3′) and mvaE-R (5′-CGCGGATCCTTATTGTTTTCTTAAATCATTTAAAATAG-3′) and pGH/mvaE as a template. The isolated *mvaE* gene fragment was excised using *NcoI* and *BamHI*, followed by insertion into the corresponding sites of pACYCDuet-1 or pYJM8 to create pYJM15 and pYJM18 respectively. The *mvaS* gene was obtained by PCR using the primers mvaS-F (5′-CCAGAGCTCAGGAGGTAAAAAAACATGACAATTGG GATTGATAAAATTA-3′) and mvaS-R (5′-CAACTGCAGTTAGTTTCGATAAGAGCGAA CG-3′) and pGH/mvaS as a template. The product of *mvaS* was introduced behind the *mvaE* gene of pYJM15 or pYJM18 after restriction with *SacI* and *PstI* to create pYJM16 (Fig.2A) and pYJM20 (Fig.2C).

### Construction of Plasmid for Lower Pathway of MVA

Plasmid pYJM14 was constructed on pTrcHis2B by introducing the *ERG8*, *ERG12*, *ERG19* and *IDI1* from *S. cerevisiae*. The four genes were ligated into the plasmid pTrcHis2B using the method established in our lab [25], [32] as follows: successive substrate fragments that designed to have long overlaps with each other were mixed, denatured and annealed. Then a circle plasmid can form and be ready for transformation. The plasmid containing four genes (*ERG12*, *ERG8*, *ERG19* and *IDI1*) was named pYJM14. The correct insertions of these genes into vectors were confirmed by PCR, restriction digestion and subsequent sequencing.

### Mutation of *mvaS* Gene from *Enterococcus Faecalis*

The mutation PCR procedure was performed according to the instruction of Fast Mutagenesis System purchased from TransGen Biotech (Beijing, China). Mutagenesis mixtures contained 10×EasyPfu polymerase buffer 2.5 µl, 200 µM dNTPs, 1 ng/ul template, 500 nM each primer, and 1 µl EasyPfu DNA polymerase. The mutated *mvaS* gene as described by[33] (*mvaS_MT_*, A110G) was obtained by PCR using the primers mvaS_MT_−F (mutation underlined, 5′-CTCTTTCGAAATCAAGGAAGGTTGTTACGGAGC-3′) and mvaS_MT−_R (5′-CTTCCTTGATTTCGAAAGAGCGAGCGAAAG-3′) and pGH/mvaS as a template. The product of *mvaS_MT_* was cloned into the plasmid pYJM15 or pYJM18 behind the *mvaE* gene after restriction with *SacI* and *PstI* to create pYJM17 (Fig.2B) and pYJM21 (Fig.2D) respectively. The pYJM17 and pYJM21 were transformed into the BL21(DE3) competent cell for expression.

### MVA Quantification by Gas Chromatography (GC)

The mevalonate produced by the engineered strains was quantitatively analyzed by GC-FID as described previously [34]. The *E. coli* strain was inoculated in 50 ml fermentation medium containing 34 µg/ml Cm resistance and incubated at 37°C and 180 rpm. When OD_600_ of the bacterial culture reached 0.6, the culture cells were induced by IPTG at a final concentration of 0.5 mM for 24 h. After fermentation broth was centrifuged for 10 min at 12000 rpm at room temperature, the supernatant was adjusted to pH 2.0 with 3 M HCl and incubated at 45°C for 1 h to convert mevalonate to mevalonic acid lactone. Then this solution was saturated with Na_2_SO_4_, and extracted with ethyl acetate. The ethyl acetate phase was transferred to a clean glass vial and dried by vacuum distillation. The residues were re-dissolved in 1 ml of ethyl acetate and analyzed by GC.

GC analysis was performed on an Agilent 7890A equipped with a flame ionization detector (FID) and a HP-AL/S column (25 m×320 µm×8 µm). N_2_ was used as carrier gas with a linear velocity of 1 ml/min. The column temperature profile was 75°C for 0.5 min, 25°C/min to 150°C, 15°C/min to 200°C, 30°C/min to 250°C, and 250°C for 5 min. The product was characterized by direct comparison with an authentic standard (Sigma-Aldrich, USA). The peak area was converted to MVA concentration by comparing with a standard curve plotted with a set of known concentration of MVA.

### Shake-flask Cultures

Shake-flask experiments were carried out in triplicate series of 600 ml sealed shake flasks containing 50 ml fermentation medium as described above plus 34 µg/ml Cm and 100 µg/ml Amp. *E. coli* strains were inoculated to the culture broth and incubated in a gyratory shaker incubator at 37°C and 180 rpm. When OD_600_ reached 0.6, IPTG was added to final concentration of 0.5 mM, and culture was further incubated at 30°C for 24 h. Then 1 ml gas sample from the headspace of the sealed cultures was analyzed as described earlier [35] using a GC (Agilent 7890A, America) equipped with a flame ionization detector (FID) and a HP-AL/S column (25 m×320 µm×8 µm). To identify bacterial isoprene production, peak retention times and mass spectra were compared with that of standard. Concentrations of isoprene produced by bacterial cells were calculated by converting GC peak area to mg of isoprene via a calibration curve. Isoprene standard (TCI-EP, Tokyo, Japan) of various concentrations was added to 600 ml fermentation medium to make a calibration curve.

### Fed-batch Fermentation

The strain was grown overnight at 37°C in 100 ml of M9 minimal media (containing K_2_HPO_4_ 1 g, Na_2_HPO_4_·12H_2_O 15.3 g, KH_2_PO_4_ 3 g, NH_4_Cl 1 g; NaCl 0.5 g, MgSO_4_ 0.5 mmol in 1 L with glucose (20 g/L) as the primary carbon source). These cultures were used to inoculate a 5-L fermentor (BIOSTAT Bplus MO5L, Sartorius, Germany) containing 3 L fermentation medium. The temperature was controlled at 30°C; the pH was maintained at 7.0 via automated addition of ammonia, and Antifoam 204 was used to prohibit foam development. The stirring speed was first set at 400 rpm and then associated with the dissolved oxygen (DO) to maintain a DO concentration of 20% saturation. The expression of plasmid-borne exogenous gene(s) for isoprene production was initiated at an OD_600_ of 12 by adding IPTG to the final concentration of 0.5 mM and inducer was added every 8 h. During the course of fermentation, the residual glucose was measured using a glucose analyzer (SBA-40D, China) and maintained below 0.5 g/l by feeding solution containing 800 g/L of glucose at appropriate rates. Then isoprene accumulation was measured every 15 min by GC as described [35]. At the same time, the growth of the bacterial culture was determined by measuring the OD_600_ with a spectrophotometer (Cary 50 UV-Vis, Varian).

## Results and Discussion

### Characterization of the Rate-limiting Step of MVA Pathway

In our previous work, a novel pathway for production of isoprene was established by assembling the whole MVA pathway derived from *S. cerevisiae* and isoprene synthase (IspS) from *Populus alba* in the *E. coli* BL21(DE3) strain. The final engineered strain YJM13 harboring the MVA pathway and *ispS_Pa_* gene could accumulate isoprene up to 2.48 mg/L and 532 mg/L under the flask and fed-batch fermentation conditions, respectively [25]. However, the yield of isoprene by the engineered strain YJM13 is too low to meet the demand for industrial application.

The low yield of isoprene might be primarily attributed to the existing of rate-limiting step of MVA pathway. In the previous study, the whole MVA pathway was divided into two parts and studied respectively: the “upper pathway”, which catalyzed the acetyl-CoA to MVA; the “lower pathway”, which converted MVA into DMAPP, and then was catalyzed by isoprene synthase into isoprene.

**Figure 3:**
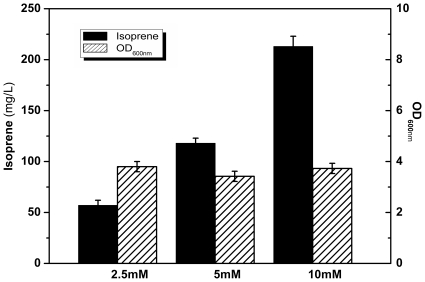
Isoprene production of Strain YJM12. The strain was cultured supplemented with different concentrations of mevalonate under flask conditions. The experiment was done in triplicate.

As for the study of the efficiency of “lower pathway”, the plasmids pYJM14 and pYJM8 were simultaneously transformed into the *E. coli* strain. To determine the extent to which isoprene production could be enhanced with increased availability of MVA, different concentrations of MVA were added to the culture broth. The maximum isoprene productions of 57, 118, 213 mg/L were obtained with the addition of 2.5 mM, 5 mM and 10 mM MVA, respectively. The cell growth has not been influenced significantly by the MVA additions (Fig. 3). The results showed that the increased isoprene productivity was in proportion to the content of mevalonate added. The maximum isoprene production of 213 mg/L was obtained with 10 mM mevalonate addition under flask condition. The results implied that the “lower pathway” was very efficient. Meanwhile, the functionality of the heterologous “upper pathway” was also under test. The results showed that overexpression of the synthetic operon of the “upper pathway” could only give a very low yield of MVA (0.026 mg/L). Based on the above-mentioned data, a conclusion could be reached that the “upper pathway” is the rate- limiting step of the whole MVA pathway.

### Evaluation of Upper Pathway of MVA from Different Origins

To eliminate the limitation of upper pathway of MVA, alteration of the origins of the “upper pathway” of MVA might be a promising way. In the previous report, the mass production of mevalonate of 47 g/L was achieved by fed-batch culture of recombinant *E. coli* harboring *mvaE* and *mvaS* genes of *E. faecalis* [36]. Yoon also demonstrated that the *mvaE* and *mvaS* genes of *E. faecalis* were the most efficient for mevalonate production among the top MVA portions used in *E. coli* [22]. Therefore, the engineered strain YJM16 containing *mvaS* and *mvaE* gene from *E. faecalis* instead of “upper pathway” from *S. cerevisiae* was constructed in this study. The strain YJM16 was inoculated in 50 ml fermentation medium and incubated at 37°C. When OD_600_ of the bacterial culture reached 0.6, IPTG was added to a final concentration of 0.5 mM, and the culture was further incubated for 24 h. MVA was isolated from fermentation broth as described method. The strain YJM16 containing the *mvaS* and *mvaE* gene from *E. faecalis* could accumulate MVA up to 1.31 g/L, which was about fifty-fold in contrast to the strain YJM11 containing *S. cerevisiae*’s upper pathway (0.026 mg/L). Obviously, the upper pathway from *E. faecalis* proves to be more effective than that from *S. cerevisiae*.

### The Effect of Mutation of *mvaS* Gene on MVA Production

The *mvaS* gene encodes the HMG-CoA synthase, the second enzyme in the MVA pathway, which catalyzes three molecules of acetyl-CoA to HMG-CoA and plays a key role in isoprenoid formation in the eukaryotic cytosol and in Gram-positive bacteria [37]. Steussy has reported that the overall reaction rate of the enzyme was increased 140-fold by mutating alanine 110 of HMG-CoA synthase from *E. faecalis* into a glycine [33]. Hence, a hypothesis would be put forward that the engineered strain with the mutated *mvaS* gene and *mvaE* from *E. faecalis* could further enhance the MVA production.

**Figure 4:**
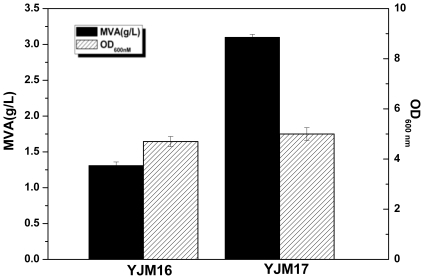
MVA production by strains with or without mutation of *mvaS* gene. The MVA isolated from cultural broth as described in “MVA quantification by gas chromatography (GC)”. The experiment was performed in triplicate.

To verify the effect of mutation of *mvaS* gene on MVA production, the recombinant strains YJM17 (*E. coli* harboring *mvaS_MT_* & *mvaE*) and YJM16 (*E. coli* containing *mvaS* & *mvaE*) were cultured in fermentation medium under shake-flask conditions. The amount of MVA accumulated in the culture media from different recombinant strains was calculated according to a standard curve plotted with a set of known concentrations of MVA. MVA concentration of the strain YJM17 reached around 3.1 g/L after being induced by 0.5 mM IPTG for 24 h, about 2.37 times higher than that of the strain YJM16 (1.31 g/L)(Fig. 4). The result demonstrated that A110G mutation of *mvaS* gene caused an appreciable increase in MVA production.

### The Effect of Optimized Upper Pathway on Isoprene Production

**Figure 5:**
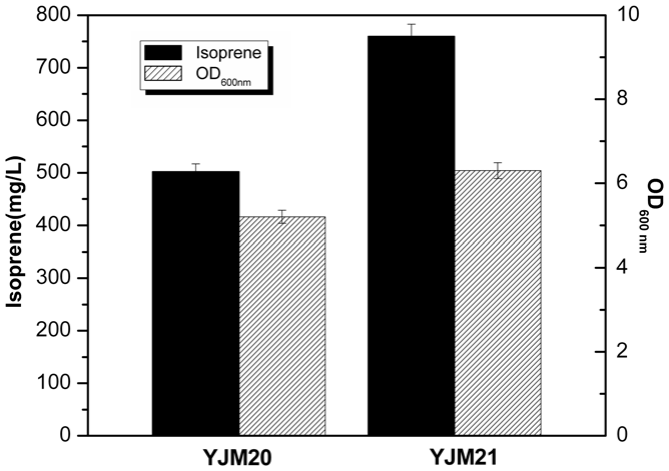
Comparison of isoprene production from different recombinant strains. The experiment was conducted under flask conditions in triplicate.

To test the function of optimized upper pathway on isoprene production, the plasmid pYJM14 containing the lower pathway from *S. cerevisiae* was transformed into the strains (*E. coli* harboring *mvaS_MT_* & *mvaE*, *mvaS* & *mvaE*), which formed the recombinant strains YJM21 and YJM20, respectively. Both of the strains YJM21 and YJM20 were inoculated into the medium under the shake-flask conditions and the cultures were induced with 0.5 mM IPTG when the OD_600_ reached 0.6–0.9. As is shown in Fig. 5, isoprene produced by the strain YJM21 reached 760 mg/L, which was about 1.5 fold to the control strain YJM20 (500 mg/L). The result showed that the optimized upper pathway played an important role in improving the isoprene production.

**Figure 6:**
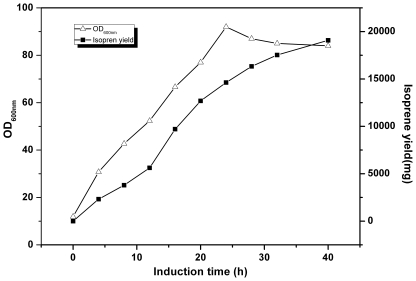
The time course of isoprene production by YJM25. Isoprene accumulation (▪) and cell growth (Δ) in YJM25, Induction was carried out at an OD_600_ of 12. Other experiment conditions were described in ‘Fed-batch fermentation’.

### Comparison of Isoprene Production in Different *E. coli* Host Strains

To choose a most efficient host strain for isoprene production, plasmids pYJM20 and pYJM21 were transformed into *E. coli* host strains JM109 (DE3), BL21 (DE3) and BL21 star^™^ (DE3) carrying plasmid pYJM14, respectively. As shown in the Table 2, BL21 star^™^ (DE3) produced more isoprene than BL21 (DE3), while the yield of isoprene produced by JM109 (DE3) was the lowest among three strains. Obviously, BL21 star^™^ (DE3) was the most suitable host to produce isoprene. No significant difference in cell growth was observed for all these strains.

**Table 2:** Isoprene production by different *E. coli* strains harboring pYJM14 and pYJM20 or pYJM14 and pYJM21 under flask conditions.

Host	Plasmids	
	pYJM14,pYJM20	pYJM14,pYJM21
*E.coli* BL21(DE3)	502 mg/L	760 mg/L
*E.coli* BL21 Start™ (DE3)	788 mg/L	1091 mg/L
*E.coli* JM109(DE3)	32 mg/L	55 mg/L

The experiment was done in triplicate.

### Fed-batch Culture of Metabolically Engineered *E. coli* Strains

To further confirm the effectiveness of the optimized MVA pathway on increasing isoprene production, fed-batch cultivation was carried out using the recombinant *E. coli* BL21^™^(DE3) strain simultaneously harboring plasmids pYJM21 and pYJM14. After depletion of the glucose added initially, glucose (800 g l^−1^) was fed and the residual glucose was maintained below 0.5 g/l to minimize acetic acid accumulation. As is shown in Fig. 6, isoprene was produced in a growth associated manner in the fed-batch phase to reach maximum concentration of 6.3 g l^−1^ after 40 h of cultivation. The conversion efficiency of glucose to isoprene in the metabolically engineered strain has attained 7%, reaching 28% of the theoretical limit (25.2%). The theoretical yield was calculated according to the following formula [13]:

1.5C_6_H_12_O_6_+2NADPH+6NAD^+^→C_5_H_8_+4CO_2_+6NADH+2NADP^+^+H_2_O+4[H].

Expressing the optimized MVA pathway and isoprene synthase from *P. alba* resulted in approximately a 12-fold increase in isoprene production compared with our previous data achieved by expression of the MVA pathway from *S. cerevisiae* and isoprene synthase in the same host strain.

In spite of the great progress for isoprene production made in *E. coli*, some problems still remained unsolved and were expected to be tackled before commercial production. For instance, the instability of the recombinant strain remains to be a severe problem due to the plasmid instability which in turn restricts the industrial application to a large extent. From metabolic engineering perspective, the stability of strain can be improved by genetic manipulation of chromosome integration technique [38], [39]. From the fermentation process aspect, immobilization of microbial cells may enhance the stability of engineered stain during the process of production [40], [41].

### Conclusions

In this paper, the efficiency of the MVA pathway on isoprene production has largely been improved by changing the source of “upper pathway” of MVA synthesis from *S. cerevisiae* to *E. faecalis* and mutating the *mvaS* gene. In the final engineered strain YJM25 (*E. coli* BL21^™^(DE3)/pYJM21, pYJM14) containing the optimized MVA pathway and isoprene synthase, it can accumulated isoprene up to 6.3 g/L after 40 h fed-batch fermentation. The conversion efficiency of glucose to isoprene (gram to gram) in the metabolically engineered strain has attained 7%, reaching 28% of the theoretical limit. These results proved to be the highest isoprene productivity reported so far [7], [29], [42].
